# Iron–Nutrient Interactions within Phytoplankton

**DOI:** 10.3389/fpls.2016.01223

**Published:** 2016-08-18

**Authors:** Hanan Schoffman, Hagar Lis, Yeala Shaked, Nir Keren

**Affiliations:** ^1^Department of Plant and Environmental Sciences, Institute of Life Sciences, The Hebrew University of JerusalemJerusalem, Israel; ^2^The Freddy and Nadine Herrmann Institute of Earth Sciences, Hebrew University of JerusalemJerusalem, Israel; ^3^Interuniversity Institute for Marine Sciences in EilatEilat, Israel

**Keywords:** nutrient, co-limitation, limitation, manganese, iron, nitrogen, photosynthesis, phytoplankton

## Abstract

Iron limits photosynthetic activity in up to one third of the world’s oceans and in many fresh water environments. When studying the effects of Fe limitation on phytoplankton or their adaptation to low Fe environments, we must take into account the numerous cellular processes within which this micronutrient plays a central role. Due to its flexible redox chemistry, Fe is indispensable in enzymatic catalysis and electron transfer reactions and is therefore closely linked to the acquisition, assimilation and utilization of essential resources. Iron limitation will therefore influence a wide range of metabolic pathways within phytoplankton, most prominently photosynthesis. In this review, we map out four well-studied interactions between Fe and essential resources: nitrogen, manganese, copper and light. Data was compiled from both field and laboratory studies to shed light on larger scale questions such as the connection between metabolic pathways and ambient iron levels and the biogeographical distribution of phytoplankton species.

## Introduction

Of all the trace metals, iron (Fe) is especially prominent in biochemical catalysis (Morel and Price, 2003; Shcolnick and Keren, 2006). Most life forms are heavily dependent on iron (for interesting exceptions see Archibald, 1983 and Aguirre et al., 2013) and phytoplankton, with their Fe-rich photosynthetic apparatus, have significantly higher Fe demands as opposed to their heterotrophic counterparts (Raven et al., 1999). The intracellular Fe content of these phototrophic microorganisms is 4–6 orders of magnitude greater than the Fe concentrations in their surroundings (Morel and Price, 2003), necessitating a substantial energetic investment in Fe uptake. Indeed, iron availability limits primary production in approximately one third of the world’s oceans (Martin, 1990; Boyd et al., 2007; Breitbarth et al., 2010) as well as some fresh water environments (McKay et al., 2004; North et al., 2007, 2008). For more on iron limitation and iron chemistry, see **Box 1**.

**Box 1.**

**Why does iron limit primary productivity in aquatic environments?** Iron limits primary productivity in up to one third of the world’s oceans. This may be surprising in light of the fact that not only is Fe the fourth most abundant element in the Earth’s crust (Taylor, 1964; Martin et al., 1989), but it is also required in trace amounts (extended Redfield ratios C:N:P is 106:16:1 as opposed to C:Fe 1:0.001; Sunda et al., 1991; Quigg et al., 2003). Unfortunately, for phytoplankton, readily bioavailable iron in aquatic environments is vanishingly scarce. Fe is found in two environmentally relevant oxidation states – Fe(II) and Fe(III). Of the pair, Fe(II) is the more soluble and reactive while Fe(III) has very low solubility and tends to precipitate as ferric oxyhydroxides (Millero, 1998; Liu and Millero, 2002). Under the oxic conditions and near neutral pH characterizing most aquatic habitats in which phytoplankton are found, iron is present in its ferric form – Fe (III). This means that dissolved Fe concentrations are low, reaching sub-nanomolar levels in open ocean waters (Johnson et al., 1997; McKay et al., 2004). In addition, over 99% of dissolved iron is bound by strong organic ligands (Gledhill and Van den Berg, 1994; Rue and Bruland, 1995; Wu and Luther, 1995), which may render the iron less bioavailable (see Shaked and Lis, 2012 and Lis et al., 2015b for discussion of this topic).

Thanks to its flexible redox chemistry, iron is well suited for electron transfer reactions and therefore serves a wide range of catalytic functions. One of the most prominent Fe-dependent processes in phytoplankton is the photosynthetic transport chain. Iron is found within the reaction center of both photosystems, with 2–3 Fe atoms in photosystem II (PSII) and 12 Fe atoms in photosystem I (PSI) (Raven et al., 1999). PSI is likely the greatest iron sink in phytoplankton. Cytochrome *c* and cytochrome *b*_*6*_*f* are two Fe-containing electron carriers within the photosynthetic and respiratory electron transfer chains. In addition, iron is a structural component of nitrate assimilation enzymes as well as in nitrogenase, the nitrogen fixing enzyme and in superoxide dismutase (Fe-SOD) which is involved in the processing of the reactive oxygen species superoxide.

Above are just some examples of iron’s essential roles and, as can be seen, Fe influences a wide range of metabolic pathways, be it directly or indirectly. Iron metabolism is thus intertwined with the metabolism of other macro and micronutrients within the cell. In this review, we explore the interactions between iron and other nutrients within phytoplankton cells, assessing iron-limiting and iron-sufficient scenarios. It should be noted that we use the term iron “limitation” to define a physiological status in which Fe is in short supply but not completely absent within the cell. The understanding of Fe–nutrient interactions at the cellular level serves to elucidate larger scale questions such as elemental stoichiometry within cells, phytoplankton community composition and interactions within natural waters and the biogeographic distribution of different phytoplankton species.

## Selected Examples of Iron Interaction With Macro and Micronutrients

### Iron/Nitrogen

**FIGURE 1:**
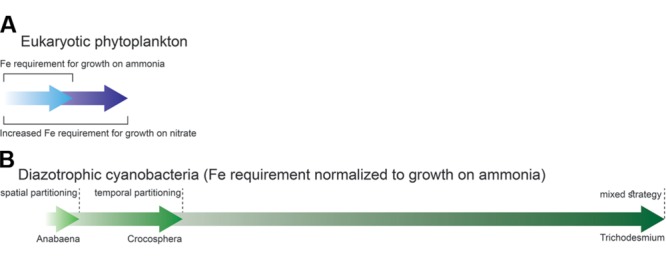
**Phytoplankton iron requirements vary as a function of the nitrogen species acquired. (A)** In eukaryotic phytoplankton nitrate assimilation requires roughly 1.8 times more iron than ammonia utilization (Raven, 1988). **(B)** In nitrogen-fixing cyanobacteria iron requirement differs as a function of the strategy utilized by the organism to partition N_2_ fixation from oxygen evolution during photosynthesis. The data in this figure is based on Raven (1988) and Berman-Frank et al. (2007).

Iron metabolism intersects with a range of macronutrients, including phosphorous (Mills et al., 2004), nitrogen and silica in diatoms (Armbrust, 2009). Here we focus on nitrogen, where interaction with iron has been extensively studied as compared to the other nutrients listed above. Theoretical calculations (Raven, 1988; Morel et al., 1991), laboratory (Maldonado and Price, 1996; Wang and Dei, 2001) and field experiments (Price et al., 1991) show that the iron requirements of phytoplankton are strongly influenced by their nitrogen source. This is because ammonia (${\mathrm{NH}}_{4}^{+}$) can be directly incorporated into amino acids, but nitrate (NO_3_) and nitrogen (N_2_) must be reduced to ammonia prior to assimilation. Phytoplankton growing on ammonia have lower Fe requirements than the same cells growing on nitrate or those which are able to convert N_2_ to ${\mathrm{NH}}_{4}^{+}$ via nitrogen fixation (**Figure 1**).

The link between iron and nitrogen fixation has received much attention: while enrichment experiments show that nitrogen may be limiting in certain marine environments over short time scales, nitrogen fixation increases the N inventory of the ocean over geological time scales (Falkowski, 1997; Mills et al., 2004). The “ultimate” limiting nutrient in large areas of the world’s oceans is thus the one that limits nitrogen fixation.

#### Examples from Eukaryotic Phytoplankton: Fe and Nitrate Assimilation

Growth on nitrate requires more iron than does growth on ammonia (**Figure 1A**). The extra iron is needed for nitrate assimilation. Firstly, the two ${\mathrm{NO}}_{3}^{–}$ reducing enzymes (nitrate and nitrite reductase) contain Fe cofactors. Secondly, the reducing power for ${\mathrm{NO}}_{3}^{–}$ reduction is produced by the photosynthetic electron transport chain. Field experiments performed by Price et al. (1991) and Ditullio et al. (1993) suggest that iron availability may limit nitrate uptake in natural waters. These studies observed increased ${\mathrm{NO}}_{3}^{–}$ uptake rates by phytoplankton following addition of iron to waters from the Equatorial Pacific and North Pacific Ocean, respectively. Indeed, the nitrate and nitrite reductase activities of eukaryotic phytoplankton decrease when cells are iron limited (Timmermans et al., 1994; Milligan and Harrison, 2000). Transcriptional analysis of *Phaeodactylum* diatoms under Fe limitation revealed a reduced capacity for nitrate uptake – genes associated with nitrate assimilation were downregulated, including the aforementioned reductase enzymes (Allen et al., 2008). Despite lowered enzyme activity, the cellular nitrogen requirement of the diatoms was still fully met and intracellular carbon to nitrogen ratios did not increase under these conditions, as would be expected under nitrogen stress. Therefore, Fe limitation will probably not lead to nitrogen limitation of the cell. Having said this, when cellular iron levels are low, phytoplankton cells do not take up nitrate as efficiently as they would under Fe-sufficient conditions. The major reason for this is an energy constraint. Iron limitation induces a decrease in the photosynthetic activity, thereby decreasing the reductive power available for NO_3_ assimilation (Morel et al., 1991; Milligan and Harrison, 2000). Therefore, iron limits nitrate acquisition primarily through its role in the photosynthetic transport chain rather than its role in nitrate-reducing enzymes. Interestingly, phytoplankton cultures grown on NO_3_ demonstrate a greater light dependency than those grown on NH_4_ under both Fe sufficient and deficient conditions (Larsson et al., 1985; Rueter and Ades, 1987; Li et al., 2004). Like iron, low light levels induce an energy constraint on nitrate assimilation because of lower photosynthetic activity. Therefore, low light can greatly aggravate the effect of Fe limitation on nitrate acquisition, a situation which may be of environmental relevance in high latitude, low iron waters.

#### Examples from Cyanobacteria: Fe and Nitrogen Fixation

Nitrogen fixation places an additional Fe burden on diazotrophic organisms on top of their routine cellular Fe requirements (Raven, 1988; Kustka et al., 2002). The N_2_-fixing enzyme nitrogenase contains 38 Fe atoms per holoenzyme (Raven, 1988; Whittaker et al., 2011). Adding to the Fe burden are the facts that: (a) nitrogenase is characterized by slow reaction rates, necessitating a relatively large intracellular pool of this enzyme and (b) N_2_ fixation requires a large amount of photosynthetically derived energy and reducing power (Raven, 1988). Nitrogenase is irreversibly inhibited by molecular oxygen (Postgate, 1998; Bergman, 2001; Gallon, 2001; Berman-Frank et al., 2003). Therefore, N_2_ fixation in diazotrophic cyanobacteria presents a unique constraint – conducting an oxygen sensitive process within an O_2_ evolving organism. This has led to the temporal and/or spatial partitioning of N_2_ fixation from photosynthetic processes within these organisms. Each partitioning strategy has its particular Fe costs (**Figure 1B**).

Unicellular marine diazotrophs, like *Cyanothece*, exhibit temporal separation of photosynthesis and N_2_ fixation with the former being performed during the day and the latter at night (Reddy et al., 1993; Colón-López et al., 1997; Welkie et al., 2014). Some filamentous cyanobacteria such as *Anabaena* implement spatial partitioning of N_2_ fixation within specialized heterocyst cells (Haselkorn, 1978; Kumar et al., 2010). The photosynthetic transport chain within heterocysts has been modified to minimize O_2_ concentrations. The oxygen evolution capacity of PSII is eliminated – some studies report the complete absence of PSII while others describe modified PSII which lack water-splitting capabilities (for further details on this fascinating subject see Wolk et al., 1994 and references therein and Cardona et al., 2009). A further modification of the photosynthetic transport chain within heterocysts is the abundance of PSI and ATP synthase (Cardona et al., 2009). PSI is situated at a bioenergetics crossroad: electrons leaving PSI can be used for linear electron flow producing ATP and NADPH or to the cyclic or pseudo-cyclic Mehler reaction which produces only ATP (Asada, 2006). The Mehler pathway may also serve as a sink for molecular oxygen within heterocysts (Berman-Frank et al., 2001b; Milligan et al., 2007). Light induced a threefold increase in O_2_ consumption by purified heterocysts cells from *Anabaena* (Milligan et al., 2007). The Mehler reaction also plays an important role in the prominent non-heterocystous marine diazotroph *Trichodesmium*. Milligan et al. (2007) show that the Mehler reaction can consume as much as 75% of gross O_2_ production when grown on N_2_ as opposed to 10% when the cells are grown on NO_3_. This can be put down to the fact that *Trichodesmium* perform both N_2_ fixation and oxygenic photosynthesis within the same cell. Both processes occur in different regions of the same cell (spatial separation) and their respective activities peak at different times during the photoperiod (temporal separation) (Berman-Frank et al., 2003). N_2_ fixation peaks around mid-day and concurs with a depression in carbon fixation, lowered photosynthetic quantum yield, decreased net oxygen evolution and an increase in the Mehler reaction (Berman-Frank et al., 2001b). The high iron content of *Trichodesmium* have been confirmed by several laboratory studies (e.g., Rueter et al., 1992; Berman-Frank et al., 2001a; Webb et al., 2001; Fu and Bell, 2003; Chappell and Webb, 2010). However, as will be discussed below, applying theoretical calculations to the question of whether iron limits nitrogen fixation the field may prove to be tricky.

The environmental distribution of the N_2_-fixation strategies described above is strongly influenced by ambient iron concentrations. Heterocystous strains such as *Anabaena* are generally found in high Fe environments such as fresh, brackish or coastal waters. These organisms are filamentous and large and are thus likely to practice luxury iron uptake and storage. However, because of their size and iron requirements, heterocystous cyanobacteria are prone to iron limitation in environmental and laboratory settings. In comparison, smaller unicellular diazotrophs such as *Cyanothece* have the advantage of a large surface area to volume ratio and can be found in open ocean waters where Fe concentration are considerably lower (Zehr et al., 2001). *Cyanothece* practices a cost-efficient method of diurnal separation of N_2_ from photosynthesis (Reddy et al., 1993; Stöckel et al., 2008) which allows for Fe recycling between photosynthetic apparatus during the day and nitrogenase at night (Tuit et al., 2004; Berman-Frank et al., 2007). The non-heterocystous open ocean diazotroph *Trichodesmium* may inhabit Fe-poor waters despite high iron demands. This is by virtue of their high Fe use efficiency (Berman-Frank et al., 2007), symbiotic association with bacteria which may aid in iron acquisition (Achilles et al., 2003; Roe et al., 2012) and the ability to access unconventional iron sources such as the colloidal and particulate pools (Rueter et al., 1992; Rubin et al., 2011; Bergman et al., 2013).

The extent of Fe limitation of environmental N_2_ fixation is still under debate. Both iron and phosphorous may limit nitrogen fixation. A recent study by Snow et al. (2015) highlights the complexity of the interactions between iron, phosphorous and diazotrophs within the Northern Atlantic. Enrichment experiments in natural environments give contrasting results: Moore et al. (2009) suggest that large-scale patterns of N_2_ fixation in the north and south Atlantic correlate primarily with Fe availability. On the other hand, Sañudo-Wilhelmy et al. (2001) showed that N fixation rates of *Trichodesmium* species in the central Atlantic Ocean are independent of dissolved iron concentrations and are rather limited by phosphate. To complicate matters further, Mills et al. (2004) report on Fe–P co-limitation of N fixation in the eastern tropical North Atlantic while Bonnet et al. (2008) observed no response to Fe and P additions in the southern subtropical Pacific Gyre.

### Iron/Manganese

The crucial roles of manganese in photosynthesis and growth of autotrophic organisms have long been recognized (Raven, 1990; Merchant and Sawaya, 2005; Nelson and Junge, 2015). Four manganese atoms form the core of the water-splitting catalytic site of PSII. Aside from this, Mn and Fe may act as cofactors in the scavenging of reactive oxygen species (ROS), with phytoplankton typically containing both Fe and Mn forms of the superoxide-disproportionating enzyme superoxide dismutase (SOD) (see Wolfe-Simon et al., 2005 for a review). Although both Fe and Mn play major roles in photosynthesis, the consequences of Fe limitation on growth and photosynthetic activity of phytoplankton are more severe than those induced by Mn limitation (Brand et al., 1983; Bruland et al., 1991; Salomon and Keren, 2011, 2015; Hernandez-Prieto et al., 2012; Fraser et al., 2013; Sharon et al., 2014; Rudolf et al., 2015). Indeed, Fe is more commonly a limiting factor on growth and photosynthetic activity of phytoplankton in natural environments. Insufficient Fe in the cell may lead to a shortage in electron carriers within the photosynthetic chain. This, in turn, results in a lack of sinks for the electrons generated by PSII activity. “Stray” electrons then leak out and reduce molecular oxygen molecules leading to the formation of the ROS – superoxide (Asada, 2006).

#### Examples from Eukaryotic Phytoplankton: Mn and Fe SOD

Studies conducted on the fresh water *Chlamydomonas reinhardtii* (Allen et al., 2006) and marine *Thalassiosira* diatom species (Peers and Price, 2004) show an increased Mn requirement and concurrent increase of Mn-SOD activity under Fe-limiting conditions. This phenomenon can be appreciated on two levels. First, Mn-SOD is necessary to deal with the ROS induced by iron limitation. Second, Mn-SOD may replace Fe-SOD. The two SOD isoforms are typically found in one of two organelles – Mn-SODs serve in the mitochondria while Fe-SODs are generally found in the chloroplast. Interestingly, Allen and coworkers (Allen et al., 2006) report the localization of Mn-SOD in both the chloroplast and mitochondria under Fe-limitation, indicating an Mn-mediated compensation for Fe limitation.

Peers and Price (2004) observed a unique photosynthetic and growth response upon addition of either Fe or Mn to cultures limited by both nutrients and a synergistic effect when adding both trace elements together. This suggests that Mn–Fe interaction extends beyond simple substitution within the SOD enzyme. Both manganese and iron limitation reduce photosynthetic yield. However, their influence is mediated via different molecular routes. While Mn availability determines the photochemical activity of the overall pool of PSII, Fe availability affects all of the membrane complexes involved in photosynthetic and respiratory electron transport. Therefore, addition of either trace metal will increase photosynthetic yield and growth but only the addition of both will result in maximal growth rates and photosynthetic activity observed in non-limited cultures.

#### Examples from Cyanobacteria: Mn Mediation of Fe Stress Response and Fe/Mn Transport

The model cyanobacterium *Synechocystis* sp. PCC 6803 show reduced photosynthetic yield under both Fe and Mn limitation (Sandstrom et al., 2002; Singh and Sherman, 2007; Shcolnick et al., 2009; Salomon and Keren, 2011). Interestingly, the study of Mn/Fe co-limitation introduced a temporal element into the interactions of these two metals (Salomon and Keren, 2015). *Synechocystis* cells acclimated to Mn deficiency prior to Fe limitation showed an atypical response to iron stress. While these cells were able to respond to iron insufficiency by inducing specific Fe transporters, other physiological responses to Fe limitation were not observed, including loss of additional PSI activity and induction of *isiAB* transcription – a hallmark of iron stress in cyanobacteria. This suggests that acclimation to environmentally relevant Mn concentrations, much lower than those employed in laboratory experiments suggest, modulates iron stress responses. At low extracellular Mn concentrations the number of functional PSII units decreases and with it the electron flux generated by water splitting activity. This may protect photosynthetic organisms from the deleterious effects of oxidative damage during Fe-limiting conditions in environments prone to fluctuations in iron concentrations.

Sharon et al. (2014) report reduced intracellular Mn content under iron limitation in *Synechocystis* sp. PCC 6803. The cause of this is hypothesized to be reduced transport of Mn into the cell. Since Mn^2+^ and Fe^3+^ have very similar coordination spheres (Williams and Frausto da Silva, 2001), it is reasonable to assume that Fe^3+^ transporters will serve as a low affinity Mn^2+^ transport system. Examination of the changes in Fe transporter expression during transition into Fe limitation show little changes in the transcription of Fe(III) permeases and an upregulation of genes encoding for proteins involved in Fe^2+^ uptake via reduction (Kranzler et al., 2014). Strong support for this hypothesis is provided by radioactive Fe transport assays (Kranzler et al., 2011) where Fe limited cells exhibit a ∼7 fold increase in the rate of reduction and a two order of magnitude increase in the rate of Fe^2+^ uptake, as compared to replete cells. Fe^2+^configuration differs from that of Fe^3+^ and Mn^2+^. Therefore, Fe^3+^ but not Fe^2+^ transport, will be able to support non-specific Mn^2+^ transport. Mn transport via Fe^3+^ transporters may account for the unidentified low affinity Mn transport system detected by Bartsevich and Pakrasi (1996). Such “promiscuity” of metal binding proteins has been demonstrated for Mn/Cu/Zn and Cu/Co (Tottey et al., 2008; Foster and Robinson, 2011).

### Iron/Copper: Plastocyanin/Cytochrome *c*_*6*_ and Copper Dependent Iron Uptake

Enrichment experiments conducted by Coale (1991) and Peers et al. (2005) in the Fe-limited Bering Sea showed that Fe addition elicited the greatest growth response, and addition of Cu induced a lower, yet significant, increase of phytoplankton growth. The addition of both trace metals had a synergistic effect on growth rates (Peers et al., 2005). Copper, like iron, plays a role in photosynthetic and respiratory electron transfer reactions. In the exploration of Cu–Fe interactions within the cell, we will consider the following biochemical intersections: (1) interchangeability between the copper-based electron carrier plastocyanin and its functionally equivalent iron based homologue – cytochrome *c*_*6*_ within the photosynthetic electron transfer chain and (2) the role of copper in high affinity iron transport systems.

Both cytochrome *c*_*6*_ (Fe based) and plastocyanin (Cu based) transfer electrons between the cytochrome *b*_*6*_*f* complex (hereafter cytochrome *b*_*6*_*f*) and PSI. In both cyanobacteria and green algae the expression of these proteins was found to be dependent on the Cu status of the cell with plastocyanin being replaced by cytochrome *c*_*6*_ under Cu-deplete conditions (Wood, 1978 and review in Merchant and Helmann, 2012). The expression of these two electron carriers is regulated as a function of copper and iron availability. For example, oceanic diatoms constitutively express the Cu-based carrier plastocyanin in lieu of the Fe-based cytochrome *c*_*6*_ (Peers and Price, 2006). The selective pressure favoring plastocyanin is a result of continual exposure to Fe limitation in open ocean environments. While lowering Fe dependency, this apparent tradeoff elevates Cu demand by 10-fold and leaves open ocean diatoms more susceptible to Cu limitation than their coastal counterparts (Peers et al., 2005). These findings could account for the dual limitation by Cu and Fe observed in the Bering Sea (Peers et al., 2005; Peers and Price, 2006; Annett et al., 2008).

Cu–Fe interactions can also take the form of biochemical dependence in certain organisms – when one nutrient is required in the other’s acquisition. A multi-copper oxidase-permease complex plays a role in high affinity Fe transport in iron-stressed eukaryotic phytoplankton, among them *C. reinhardtii* (La Fontaine et al., 2002; Merchant et al., 2006; Blaby-Haas and Merchant, 2013) and several marine diatoms (Maldonado et al., 2006). As with plastocyanin expression, this system also leads to an increased cellular Cu demand under Fe limitation. Since copper supports increased iron uptake, the addition of both trace metals to co-limited cells induces a synergistic growth response. Interestingly, a second well-characterized high affinity iron transport system within phytoplankton also features Cu–Fe interplay. Some Fe-limited cyanobacteria produce low-molecular-weight Fe-specific chelators known as siderophores. Along with their high specificity for binding iron, siderophores are also able to bind copper, a potentially harmful trace metal (Sunda, 1975). Several studies have suggested that siderophores may mitigate copper toxicity (the Cu-siderohore complex is not toxic to cells and cannot be transported into the cell); however, this is a benefit second to their primary role in iron acquisition (Clarke et al., 1987; Nicolaisen et al., 2010).

### Iron/Light

**FIGURE 2:**
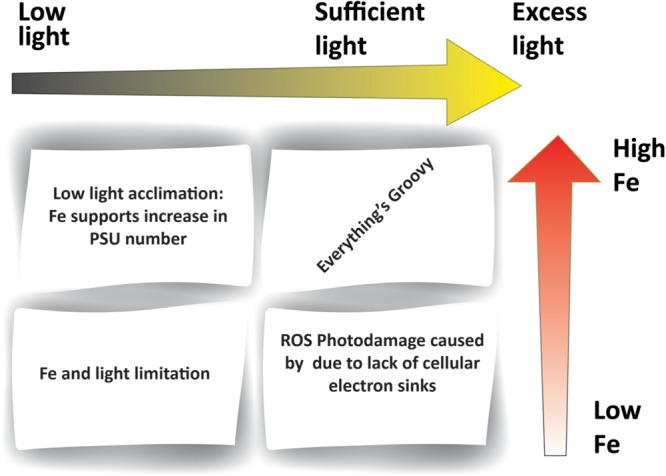
**Iron–light interactions within phytoplankton.** The figure outlines different combinations of iron and light availability. Low iron (e.g., in HNLC regions) results in insufficient Fe-containing electron carriers within the photosynthetic electron transport chain. Low light levels (e.g., high latitudes) induce an increase in Fe requirements in order to support greater PSU number. A lack in both Fe and light (e.g., high latitude HNLC regions) results in the dual limitation of photosynthesis.

Light energy imposes a fundamental constraint on primary productivity; and given the strong coupling between iron and photosynthetic electron transfer, Fe-light interactions are inevitable (**Figure 2**). Sub-optimal light conditions need not be confined to low light intensities but can also include damaging high light intensities. Both have been shown to inhibit the photosynthetic activity and the growth of phytoplankton (Adir et al., 2003; Hakala et al., 2005; Komenda et al., 2012; Tyystjärvi, 2013; Roach and Krieger-Liszkay, 2014; Järvi et al., 2015). The definition of low, high and excess light is organism dependent, with different strains exhibiting unique responses to a range of light intensities. These can be described by the photosynthesis vs. irradiation (PI) curve under defined conditions (Hassidim et al., 1997; Eisenstadt et al., 2008).

#### Examples from Eukaryotic Phytoplankton: PSI and Cytochrome *b_*6*_f*

Studies of phytoplankton cultures have long shown a discrepancy in the iron requirements and use efficiencies (expressed as mol assimilated C per mol cellular Fe) of coastal as opposed to open ocean eukaryotic phytoplankton (Sunda and Huntsman, 1995, 1997) and cyanobacteria (Sunda and Huntsman, 2015). This phenomenon may be explained by differences in the photosynthetic architecture of organisms residing in these two environments. Oceanic diatoms contain several fold less PSI and cytochrome *b_*6*_f* as compared to coastal diatoms (Strzepek and Harrison, 2004). Both these components of the photosynthetic apparatus are major sinks of cellular iron (Raven et al., 1999), resulting in lower Fe requirements of open ocean as compared to coastal diatoms. Such differences most likely reflect the evolution of oceanic phytoplankton under Fe-poor but relatively uniform light conditions. Strzepek and Harrison (2004) show that while oceanic isolates have a much lower Fe requirement due to a lowered PSI:PSII and cytochrome *b*_*6*_*f*:PSII ratios, this also causes a reduced tolerance to rapid changes in irradiance as compared to coastal isolates. On the other hand, coastal diatoms have a greater Fe requirement but are equipped to handle the fluctuating light intensities which characterize dynamic and turbid coastal waters. This is because PSI and cytochrome *b*_*6*_*f* can participate in cyclic electron transport processes, which alleviate excitation pressure (Takahashi et al., 2013).

The capture of photons by any single photosystem can be considered as a statistical event. Therefore one way of acclimating to sub-saturating light conditions, is to increase the number of active photosynthetic units (PSU). This, in turn, increases the iron requirement of cells (Falkowski et al., 1981; Raven, 1990). Culture experiments show increased Fe requirements of phytoplankton grown under low light intensities (e.g., Sunda and Huntsman, 1997, 2011, 2015). Therefore, environments characterized by both low light intensities and paucity of iron very likely foster the dual limitation of photosynthetic organisms by iron and light. One striking example relates to high latitude High Nutrient Low Chlorophyll (HNLC) regions distinguished by low Fe concentrations in ambient waters and seasonally low light conditions. Incubation experiments in these areas show that the increase of irradiance and addition of Fe elicited a growth response independently while a concomitant increase in both factors acted synergistically to produce the greatest growth response (Maldonado and Price, 1999). These results can be explained by a biochemical dependence of light on iron – a situation in which one resource in required in order to acquire another resource efficiently. A recent study on Southern Ocean diatoms uncouples the traditional association between low light and high iron demand (Strzepek et al., 2012). These organisms were able to increase their light use efficiency without increasing their iron demands significantly. This is accomplished by increasing PSU size rather than the number of PSUs, a strategy that is not as costly in iron.

#### Examples from Cyanobacteria: *isiAB*

Typical cyanobacterial Fe-stress responses include a decrease in both PSI abundance (Fraser et al., 2013) and phycobillisome cross-section (Sherman and Sherman, 1983; Sandmann, 1985; Salomon and Keren, 2015) and, in certain strains, the expression of *isiAB* (Laudenbach and Straus, 1988; Ghassemian and Straus, 1996; Park et al., 1999; Dühring et al., 2006). Lowering the number of PSI complexes reduces Fe requirements as does the induction of *isiB* coding for a flavodoxin that can replace ferredoxin within the electron transport chain (Laudenbach et al., 1988). The function of IsiA, on the other hand, remains ambiguous (Singh and Sherman, 2007; Behrenfeld and Milligan, 2013). IsiA bears a strong structural similarity to the PSII chlorophyll binding CP43 protein (reviewed in Singh and Sherman, 2007). IsiA binds chlorophyll and forms a ring around photosystem I (Bibby et al., 2001b; Boekema et al., 2001). However, it can also form aggregates in the total absence of PSI (Yeremenko et al., 2004; Ihalainen et al., 2005; Wilson et al., 2007; Berera et al., 2009). This protein has been suggested to offset either of the following possible outcomes of iron-light limitation in photosynthetic organisms: (1) a decrease in photosynthetic activity and (2) potential oxidative damage under high light conditions arising from a shortage in Fe electron carriers.

It has been suggested that IsiA increases the PSU size and PSI cross-section under Fe deficiency in order to compensate for the lowering of phycobilisome and PSI levels (Bibby et al., 2001a; Fromme et al., 2003; Ryan-Keogh et al., 2012; Sun and Golbeck, 2015). On the other hand, during iron starvation electron flux originating from PSII is likely to cause oxidative damage due to lack of Fe-based electron carriers. Indeed, several reports suggests that IsiA functions in the quenching of excitation energy that cannot be utilized by the organism under Fe stress, thereby protecting PSII from oxidative damage (Park et al., 1999; Sandstrom et al., 2002; van der Weij-de Wit et al., 2007; Berera et al., 2009; Wahadoszamen et al., 2015). A third, but not exclusive, role for IsiA is as a reservoir for chlorophyll which may balance the degradation of PSI during iron recycling when cells are iron limited (Riethman and Sherman, 1988). Chlorophyll life-time may exceed that of the proteins which bind it (Vavilin et al., 2005). In such cases when the binding proteins are degraded chlorophyll must be bound in a way that would prevent photodynamic damages.

## Summary

**FIGURE 3:**
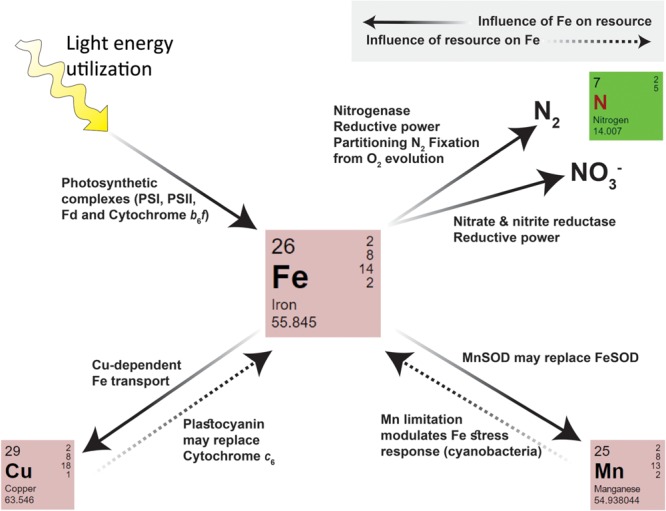
**A summary of intracellular interactions between iron and N, Mn, Cu, and light within phytoplankton.** Iron influences the requirement and uptake/utilization of a given resource (indicated by arrows with solid lines). The Fe requiring processes and enzymes of each pathway are indicated next to the arrow. Iron itself may also be influenced by other resources (indicated by arrows with dashed lines).

Iron plays a central role in energy production and biochemical catalysis within phytoplankton cells, being involved in multiple metabolic pathways either directly, via its catalytic role in enzymes, or indirectly via its part in the production of energy rich molecules such as NADPH and ATP. Fe thus influences the requirements, acquisition and utilization of essential resources within phytoplankton cells. These same resources, in turn, modulate iron requirements, acquisition and use efficiency (summarized in **Figure 3** and **Table 1**). Considering the core functions iron fulfills in cell metabolism and its relative scarcity in aquatic environments, it is little wonder that the availability of this trace metal exerts strong selective pressure on phytoplankton. Under iron-limiting conditions, phytoplankton must find a way to increase cellular iron on the one hand and decrease their Fe demands on the other. Both types of adaptation are closely tied in to light utilization and the metabolic pathways of different nutrients within the cell. **Table 1** summarizes adaptations to low Fe and the tradeoffs which may occur within organisms from the perspective of nitrogen, manganese and copper metabolisms as well as light utilization.

**Table 1:** Tradeoffs and co-limitation scenarios for iron-nitrogen, manganese, copper, and light interactions.

Resource	Description of interaction	Type of interaction/s with iron	Trade offs	Adaptations to low [Fe]	Co-limitation scenarios
Nitrogen	Photochemically produced reductive energy (NADPH) requires Fe based electron carriers. Fe dependent enzymes: Nitrate and nitrite reductases (NO_3_) Nitrogenase (N_2_)	Biochemical dependence	Ambient Fe concentrations constrain biogeographic distribution of diazotrophic/non-diazotrophic phytoplankton in accordance with their partitioning strategy.	Temporal partitioning of N_2_ fixations allows for smaller cells and Fe recycling.	While iron limitation lowers the efficiency of NO_3_ uptake, nitrogen demands of Fe-limited cells can still be fully met. Nitrogen fixation may be Fe limited.
Manganese	Mn-SOD may replace Fe-SOD Transporter promiscuity – Mn may enter cell via specific Fe transporters.	Substitution Indirect		Mn-SOD compensates for decreased levels of Fe-SOD under limitation.	Mn concentrations in most natural waters are above limiting levels (see Brand et al., 1983 and Hecky and Kilham, 1988).
Copper	Plastocyanin is a Cu based electron carrier which may substitute for the Fe containing cytochrome *c*_*6*_. Cu dependent high affinity Fe transporters.	Substitution Biochemical dependence	Fe demands decreased but greater Cu demands of open ocean diatoms leave them more prone to Cu limitation.	Plastocyanin is constitutively expressed in open ocean diatoms in lieu of cytochrome *c*_*6*_.	Ambient Cu concentrations are mostly sufficient (see Peers et al., 2005 and Peers and Price, 2006).
Light	Iron is essential to photosynthesis, the greatest cellular Fe sink.	Biochemical dependence	Species with lowered PSI:PSII and Cytochrome *b*_*6*_*f*:PSII ratios have lower Fe demands but smaller dynamic range for handling fluctuating light intensities.	Open ocean strains have less PSI and Cytochrome *b*_*6*_*f*. Southern Ocean diatoms increase PSU size rather than number, increasing light capture without inflating Fe costs.	Low iron waters at high latitudes.

One of the most prominent characteristics of iron-limited cells is a decrease in size – not only do smaller cells have smaller nutrient requirements but the resultant increase in surface area to volume ratio is of great advantage under Fe-deficient conditions (see Sunda and Huntsman, 1995; and Lis et al., 2015a for a discussion of this). Under iron limitation phytoplankton also express high affinity transport systems (Maldonado et al., 1999; Maldonado and Price, 2001; Kustka et al., 2007; Allen et al., 2008; Kranzler et al., 2014). Both these strategies aid in the acquisition of Fe when it is a limiting resource. In addition, cells can reduce their iron requirements within different metabolic pathways. This may include substitution of Fe with other catalytically active trace metals, a decrease in Fe-rich cellular components or dependence on alternative biochemical pathways. Such changes may be temporary or even permanent if a species has evolved under constant Fe stress.

A possible outcome of iron’s interaction with light and nutrients is co-limitation. Co-limitation is the synchronous limitation of growth and/or productivity by iron together with one or more resources (see Arrigo, 2005). Saito et al. (2008) describe three co-limitation scenarios, each of which is based on the mode of interaction between the two resources: (1) Independent nutrient co-limitation where two nutrients are each drawn down to a point which limits growth. (2) Biochemical substitution co-limitation where one nutrient can replace another in a bioactive molecule and (3) Biochemically dependent co-limitation where one nutrient is needed for the uptake of another nutrient. This can also be extended to the utilization of a given resource (e.g., light). It should be noted that the spatial patterns and significance of co-limitation within natural environments remain unclear (Moore et al., 2013). These depend on a great deal of factors such as nutrient supply, biological nutrient demand and community interactions including competition, symbiosis, parasitism and predation. Nonetheless, the study of Fe-nutrient/light interactions on a single cell level in a laboratory setting defines the conceptual limits for the environmental relevance of co-limitation. **Table 1** lists the iron-light/nutrient interactions discussed in this review and their potential for co-limitation within environmental setting.

The question of nutrient requirement and usage by phytoplankton is multifaceted. Nutrient supply and demand are dictated by a wide range of factors. When looking at supply, we must consider abiotic factors such as nutrient influx, chemical speciation and chemical interactions and photochemistry as well as biotic factors such as competition, symbiosis and physiological uptake systems. Demand is influenced by factors such as cell size, nutrient use efficiency, luxury uptake or storage, symbiosis, and of course, the nutrient interactions within the cell. Moreover, at both the single cell and the community level, nutrient demands are not static. They are influenced by the dynamics between cell physiology, biochemistry and environmental cues such as light. This results in distinct requirements amongst phytoplankton leading to diverse limitation patterns in different environments and the differential limitation of different phytoplankton species found within the same environment (Moore et al., 2013). As research progresses in the mapping out of the intricate network of nutrient interactions within the cell, so will our ability to understand phytoplankton dynamics within their natural environments, including nutrient limitations and growth patterns.

## Author Contributions

All authors listed, have made substantial, direct and intellectual contribution to the work, and approved it for publication.

## Conflict of Interest Statement

The authors declare that the research was conducted in the absence of any commercial or financial relationships that could be construed as a potential conflict of interest.
